# Novel Sequence Type in *Bacillus cereus* Strains Associated with Nosocomial Infections and Bacteremia, Japan

**DOI:** 10.3201/eid2505.171890

**Published:** 2019-05

**Authors:** Reiko Akamatsu, Masato Suzuki, Keiji Okinaka, Teppei Sasahara, Kunikazu Yamane, Satowa Suzuki, Daisuke Fujikura, Yoshikazu Furuta, Naomi Ohnishi, Minoru Esaki, Keigo Shibayama, Hideaki Higashi

**Affiliations:** Hokkaido University, Sapporo, Japan (R. Akamatsu, D. Fujikura, Y. Furuta, N. Ohnishi, H. Higashi);; National Institute of Infectious Diseases, Tokyo, Japan (M. Suzuki, S. Suzuki, K. Shibayama);; National Cancer Center Hospital, Tokyo (K. Okinaka, M. Esaki);; Jichi Medical University, Tochigi, Japan (T. Sasahara); Yonago Medical Center, Tottori, Japan (K. Yamane)

**Keywords:** Bacillus cereus, bacteria, nosocomial infections, bacteremia, multilocus sequence typing, pulsed-field gel electrophoresis, repetitive-element PCR, phylogenetic analysis, food safety, enteric infections, Japan

## Abstract

*Bacillus cereus* is associated with foodborne illnesses characterized by vomiting and diarrhea. Although some *B. cereus* strains that cause severe extraintestinal infections and nosocomial infections are recognized as serious public health threats in healthcare settings, the genetic backgrounds of *B. cereus* strains causing such infections remain unknown. By conducting pulsed-field gel electrophoresis and multilocus sequence typing, we found that a novel sequence type (ST), newly registered as ST1420, was the dominant ST isolated from the cases of nosocomial infections that occurred in 3 locations in Japan in 2006, 2013, and 2016. Phylogenetic analysis showed that ST1420 strains belonged to the Cereus III lineage, which is much closer to the Anthracis lineage than to other Cereus lineages. Our results suggest that ST1420 is a prevalent ST in *B. cereus* strains that have caused recent nosocomial infections in Japan.

## Abstract 2

This sequence type was dominant in isolates from bacteremia patients in 3 hospitals.

*Bacillus cereus* causes foodborne illness that is characterized by vomiting because of production of emetic toxin and diarrhea because of production of enterotoxin (*1*). In addition to foodborne illness, *B. cereus* causes severe nongastrointestinal infections, such as bacteremia (*2*), endocarditis (*3*), meningoencephalitis (*4*), and pneumonia (*5*). Severe infections occur particularly in immunocompromised patients, sometimes resulting in nosocomial infections. Such nosocomial infections with *B. cereus* have been reported to be associated with *B. cereus* contamination of ventilator equipment (*6*), intravenous catheters (*7*), and linens (*8*).

Multilocus sequence typing (MLST) is a molecular typing technique based on 7 housekeeping genes (*9*). The phylogenetic tree of isolates of *B. cereus* group species, which include *B. anthracis*, *B. thuringiensis*, and several *B. cereus* subspecies (*10*), clusters into clades 1, 2, and 3. Clade 1 consists of 4 lineages defined by Priest et al. (*9*). Previous MLST studies have shown that clinical isolates of *B. cereus* are phylogenetically diverse and clustered mainly in clades 1 and 2 (*11*), suggesting that specific clones or lineages of *B. cereus* are associated with specific illnesses and severe infections. For example, Zhang et al. showed that isolates from outbreaks of nosocomial infections were closely related to *B. anthracis* by phylogenetic analysis using MLST (*12*).

There have been only a few reports on MLST analysis of *B. cereus* isolates that have caused nosocomial infections (*12*,*13*). The objective of our study was to elucidate the genetic characteristics of *B. cereus* strains that were isolated from recent nosocomial infections in 4 hospitals in Japan during 2006, 2012, 2013, and 2016. A novel sequence type (ST) was dominant in isolates from 3 of the hospitals, suggesting a strong association of the ST with recent nosocomial infections in Japan.

## Materials and Methods

### Bacterial Strains

We used 4 groups of *B. cereus* strains in our study (Table 1; Table 2; Tables 3, 4). A total of 69 strains were isolated from equipment and patients in a hospital in Tokyo that had *B. cereus* infections in 2013. These strains were designated Tokyo strains. Two blood cultures were prepared per patient. A patient was considered to have bacteremia when a *B. cereus* strain was isolated from both cultures. A patient was designated as having pseudobacteremia when a *B. cereus* strain was isolated from only 1 of the 2 cultures. A total of 65 strains were isolated from equipment and patients in a hospital in Tochigi that had *B. cereus* infections in 2006. These strains were designated Tochigi strains (*14*). During 2012, four strains were isolated from patients in hospitals in Kochi. These strains were designated Kochi strains. During 2016, ten strains were isolated from patients in hospitals in Tottori. These strains were designated Tottori strains. The Tokyo, Tottori, and Kochi strains were isolated by medical institutions in Japan (Tokyo, Tottori, and Kochi, respectively), and the Tochigi strains were isolated by Jichi Medical University (Tochigi, Japan).

**Table 1:** Sequence types and sources of 69 *Bacillus cereus* Tokyo strains, Japan

Sequence type	Sample name	Source
1420*	Tokyo_ID2	Patient blood
	Tokyo_ID3	Patient blood†
	Tokyo_ID4	Patient blood†
	Tokyo_ID5	Patient blood†
	Tokyo_ID7	Patient blood†
	Tokyo_ID9	Patient blood
	Tokyo_ID10	Patient blood
	Tokyo_ID11	Patient blood
	Tokyo_ID12_#1‡	Patient blood
	Tokyo_ID12_#2‡	Patient blood
	Tokyo_ID14	Patient blood
	Tokyo_ID15	Patient blood
	Tokyo_ID18	Patient blood†
	Tokyo_ID19	Patient blood†
	Tokyo_ID23	Towel
	Tokyo_ID24	Towel
	Tokyo_ID25	Towel
	Tokyo_ID29	Microwave
	Tokyo_ID31	Patient blood
	Tokyo_ID32	Towel
	Tokyo_ID34	Towel
	Tokyo_ID36	Towel
	Tokyo_ID37	Towel
	Tokyo_ID46	Towel
	Tokyo_ID47	Towel
	Tokyo_ID52	Towel
	Tokyo_ID55	Towel
	Tokyo_ID57	Towel
	Tokyo_ID61	Towel
1421*	Tokyo_ID6	Patient blood†
	Tokyo_ID13	Patient blood
	Tokyo_ID16	Patient blood†
	Tokyo_ID17	Patient blood
	Tokyo_ID28	Microwave
1425*	Tokyo_ID59	Towel
	Tokyo_ID67	Washed white coat
1428*	Tokyo_ID8	Patient blood
1429*	Tokyo_D27	Towel
1430*	Tokyo_ID53	Towel
1431*	Tokyo_ID66	Washed white coat
26	Tokyo_ID22	Towel
	Tokyo_ID38	Towel
	Tokyo_ID41	Towel
	Tokyo_ID54	Towel
	Tokyo_ID56	Towel
	Tokyo_ID60	Towel
	Tokyo_ID64	Plastic bag
73	Tokyo_ID51	Towel
75	Tokyo_ID49	Towel
163	Tokyo_ID33	Towel
	Tokyo_ID35	Towel
	Tokyo_ID39	Towel
	Tokyo_ID42	Towel
	Tokyo_ID48	Towel
177	Tokyo_ID20	Patient blood†
365	Tokyo_ID21	Towel
	Tokyo_ID44	Towel
	Tokyo_ID65	Plastic bag
368	Tokyo_ID1	Patient blood
	Tokyo_ID40	Towel
	Tokyo_ID50	Towel
	Tokyo_ID58	Towel
	Tokyo_ID63	Microwave
953	Tokyo_ID26	Towel
994	Tokyo_ID45	Towel
	Tokyo_ID62	Towel
1028	Tokyo_ID68	Washed towel
	Tokyo_ID69	Towel
1050	Tokyo_ID43	Towel

*Sequence type found in this study. †Pseudobacteremia was suspected. ‡Samples isolated from the same patients at different time points.

**Table 2:** Sequences types and source of 65 *Bacillus cereus* Tochigi strains, Japan*

Sequence type	Sample name	Source
1420†	Tochigi_ID1	Infusion
	Tochigi_ID3	Infusion
	Tochigi_ID5	Infusion
	Tochigi_ID31_#1‡	Patient blood
	Tochigi_ID31_#3‡	Patient blood
	Tochigi_ID31_#4‡	Patient blood
	Tochigi_ID39	Patient blood
	Tochigi_ID41_#1‡	Patient blood
	Tochigi_ID41_#2‡	Patient blood
	Tochigi_ID48	Patient blood§
	Tochigi_ID49_#1‡	Patient blood
	Tochigi_ID49_#2‡	Patient blood
	Tochigi_ID51	Patient blood§
	Tochigi_ID52	Patient blood§
	Tochigi_ID53	Patient blood§
	Tochigi_ID75	Linen room washed towel
1421†	Tochigi_ID9	Sheet
1422†	Tochigi_ID14	ED washed sheet
1423†	Tochigi_ID28	Ward washing machine
	Tochigi_ID29	Ward washing machine
1424†	Tochigi_ID30	Ward washing machine
1425†	Tochigi_ID37_#1‡	Patient blood
	Tochigi_ID37_#2‡	Patient blood
	Tochigi_ID46	Patient blood§
1426†	Tochigi_ID58	Patient blood§
1427†	Tochigi_ID60	Patient blood§
139	Tochigi_ID21	Vender washed sheet
151	Tochigi_ID17	ED washed sheet
	Tochigi_ID23	Vender washed sheet
163	Tochigi_ID6	Patient skin
	Tochigi_ID13	ED washed sheet
	Tochigi_ID16	ED washed sheet
	Tochigi_ID20	ED washed towel
	Tochigi_ID45	Patient blood§
	Tochigi_ID55	Patient blood§
	Tochigi_ID64	Linen room washed towel
	Tochigi_ID65	Linen room washed towel
	Tochigi_ID71	Linen room washed towel
164	Tochigi_ID56	Patient blood§
167	Tochigi_ID7	Patient skin
	Tochigi_ID15	ED washed sheet
	Tochigi_ID22	Vender washed sheet
	Tochigi_ID36	Patient blood
	Tochigi_ID42	Patient blood§
	Tochigi_ID44	Patient blood§
	Tochigi_ID62	Linen room washed towel
	Tochigi_ID63	Linen room washed towel
	Tochigi_ID70	Linen room washed towel
365	Tochigi_ID2	Infusion
	Tochigi_ID4	Infusion
	Tochigi_ID35	Patient blood
	Tochigi_ID57	Patient blood§
368	Tochigi_ID19	ED washed sheet
	Tochigi_ID31_#2‡	Patient blood
	Tochigi_ID40	Patient blood
	Tochigi_ID43	Patient blood
	Tochigi_ID47	Patient blood§
	Tochigi_ID61	Patient blood§
953	Tochigi_ID12	Sheet
	Tochigi_ID18	ED washed sheet
	Tochigi_ID74	Linen room washed towel
992	Tochigi_ID11	Sheet
999	Tochigi_ID10	Sheet
	Tochigi_ID54	Patient blood§
1263	Tochigi_ID8	Sheet

*ED, emergency department. †Sequence types found in this study. ‡Samples isolated from the same patients at different time points. §Pseudobacteremia was suspected.

**Table 3:** Sequence types and source of 10 *Bacillus cereus* Tottori strains, Japan*

Sequence type	Sample name
1420†	Tottori_ID2
	Tottori_ID4_#2‡
	Tottori_ID5
	Tottori_ID6
	Tottori_ID8
1431†	Tottori_ID3
1828†	Tottori_ID1
163	Tottori_ID4_#1‡
368	Tottori_ID7
953	Tottori_ID9

*All strains were isolated from patient blood. †Sequence types found in this study. ‡Samples isolated from the same patients at different time points.

**Table 4:** Sequence types and source of 4 *Bacillus cereus* Kochi strains, Japan*

Sequence type	Sample name
1432†	Kochi_ID3
368	Kochi_ID1_#1‡
	Kochi_ID1_#2‡
427	Kochi_ID2

*All strains were isolated from patient blood. †Sequence type found in this study. ‡Samples isolated from the same patients at different time points.

### Pulsed-Field Gel Electrophoresis

We performed pulsed-field gel electrophoresis (PFGE) for the Tokyo, Tochigi, and Tottori strains to determine their genetic relatedness. DNA was digested with *Sma*I. We used the CHEF Mapper Pulsed Field Electrophoresis Systems (Bio-Rad Laboratories, https:///www.bio-rad.com) for electrophoresis. We analyzed resulting photographic images by using the GelCompar II software (Applied Maths, http://www.applied-maths.com). Isolates with a PFGE fingerprint similarity >80% were clustered into the same PFGE cluster type (*15*).

### Repetitive-Element PCR

We isolated DNA by using the Ultraclean Microbial DNA Isolation Kit (MO BIO Laboratories, https://mobio.com) and performed DNA amplification by using a DiversiLab *Bacillus* Fingerprinting Kit (bioMérieux, https://www.biomerieux.com). We separated repetitive-element PCR (rep-PCR) amplicons in a microfluidics DNA chip by using an Agilent 2100 Bioanalyzer (Agilent Technologies, Inc., https://www.agilent.com) and performed analysis by using DiversiLab version 3.4 software (bioMérieux), which uses the Pearson product-moment correlation and the unweighted pair group method with arithmetic averages. Isolates with a rep-PCR fingerprint similarity >96% were categorized as being in the same rep-PCR cluster type (*16*).

### Multilocus Sequence Typing

For the Tokyo, Kochi and Tottori strains, 7 sets of primers in the *B. cereus* MLST database (http://www.pubmlst.org/bcereus) were used to perform PCR analysis for the 7 MLST gene loci (*glpF*, *gmk*, *ilvD*, *pta*, *pur*, *pycA*, and *tpi*). We purified PCR products by using QIAquick PCR Purification (QIAGEN, https://www.qiagen.com), followed by sequencing with the 3130xl Genetic Analyzer (Life Technologies, https://www.thermofisher.com), after performing a reaction using the BigDye Terminator v3.1 Cycle Sequencing Kit (Life Technologies). Sequences of the 7 genes were trimmed to the lengths described in the database. Each unique sequence was assigned an allele number according to the *B. cereus* MLST database. We determined the ST by combining the allele numbers for all 7 loci. For the Tochigi strains, we determined STs by using results of whole-genome sequencing with the Illumina MiSeq Platform (Illumina, https://www.illumina.com). Sequences were de novo assembled by using Platanus_B version 1.1.0) (*17*). STs were determined from the de novo assembled genomes by using MLST2.0 (*18*).

We constructed phylogenetic trees by using MEGA7 (*19*) and aligned sequences produced by concatenating the sequence of each locus for MLST by using the neighbor-joining method (*19*,*20*). Branch quality was evaluated by using a bootstrap test with 1,000 replicates. We obtained sequences of each locus in the representative strains of *B. cereus*, *B. anthracis*, *B. thuringiensis*, *B. mycoides*, *B. pseudomycoides*, and *B. weihenstephanensis* from the *B. cereus* MLST database and used as references.

## Results

### Genotype Profile of Tokyo Strains

**Figure 1:**
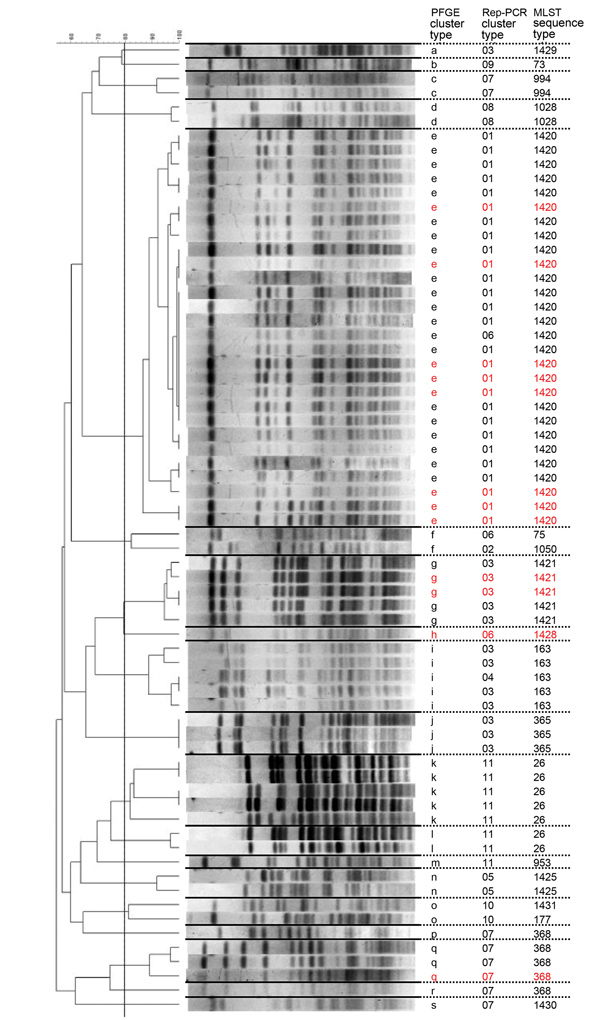
PFGE of the Tokyo strains of *Bacillus cereus* isolates, Japan. The 80% similarity cutoff for PFGE cluster typing is shown as a vertical line in the phylogenetic tree. Red letters and numbers represent samples isolated from patients with bacteremia. Rep-PCR cluster type and MLST sequence types are also shown. The Tokyo_ID31 strain was not analyzed. Scale bar indicates percent similarity. MLST, multilocus sequence typing; PFGE, pulsed-field gel electrophoresis; Rep-PCR, repetitive-element PCR.

During June–August 2013, a hospital in Tokyo had nosocomial infections attributed to *B. cereus* that caused bacteremia in 13 patients and led to the death of 2 patients. To genetically characterize *B. cereus* isolated from patients and equipment in the hospital, which we named Tokyo strains, we performed PFGE analysis and rep-PCR fingerprinting. Among the Tokyo strains, we detected 19 PFGE cluster types and 11 rep-PCR cluster types. We found by PFGE analysis that more than one third of the Tokyo strains were in a single PFGE cluster, which was denoted cluster e (Figure 1). Rep-PCR fingerprinting showed results consistent with those of PFGE analysis, in which all but 1 of the isolates in cluster e identified by PFGE were in the same cluster type identified by rep-PCR (Figure 1).

### MLST Analysis of Tokyo, Tochigi, Tottori, and Kochi Strains

To identify the genetic characteristics of the Tokyo strains, we performed MLST analysis for the Tokyo strains. For the 69 isolates, Tokyo strains had 18 distinct STs, including 7 novel STs (Table 1). The strains in the largest cluster, which was based on PFGE and rep-PCR analyses, were all determined to have the same novel ST that had been newly registered as ST1420. Therefore, results indicated the presence of a major cluster in the Tokyo strains (Figure 1).

Thirteen strains from blood samples isolated from 12 patients with bacteremia had the following 4 STs: 1420, 1421, 1428, and 368 (Table 1). Nine isolates with ST1420 were isolated from 8 of the 12 patients. We observed that strains that caused bacteremia were identified as ST1420 at a significantly high rate (p = 0.03 by Fisher exact test), suggesting an association between ST1420 and bacteremia cases with Tokyo strains.

To evaluate the relationship between ST1420 and other *B. cereus* isolates associated with nosocomial infections, we performed MLST for the Tochigi, Tottori and Kochi strains. The strains were also isolates from nosocomial infections by *B. cereus* in Japan but occurred in different prefectures and different years. The Tochigi strains were determined to have 19 distinct STs (Table 2), 8 of which were novel. Fifteen isolates from blood samples of 9 patients with bacteremia had the following 5 STs: 1420, 1425, 167, 365, and 368. Eight strains with ST1420 were isolated from 4 of 9 patients with bacteremia. The Tottori strains, all isolated from blood samples of patients with bacteremia, had the following 6 STs: 1420, 1431, 163, 368, 953, and 1828 (Table 3). ST1420 was isolated from 5 patients. ST1420 was also a dominant ST among isolates from patients with bacteremia involving the Tochigi and Tottori strains, similar to that for the Tokyo strains. The Kochi strains, which were also isolated from 4 blood samples of 3 patients with bacteremia, had the following 3 STs: 1432, 368, and 427 (Table 4). Although it was not possible to rule out the likelihood that strains with other STs were associated with the bacteremia cases in Kochi, in general, samples from different hospitals suggested a significant relationship between ST1420 and bacteremia (p = 0.0006 by Fisher exact test) even when the Kochi strains were taken into account.

ST1420 was a novel ST that had a new *tpi* allele number, registered as 210 (Table 5). ST1420 had a combination of allele numbers that was the most similar to that of ST366 in the MLST database. ST1420 and ST366 had the same allele numbers at 5 (*glpF, gmk, pta, pur*, and *pycA*) of 7 loci used in the MLST analysis. Allele *tpi* 210 of ST1420 differed from *tpi* allele 83 of ST366 by only 1 nt. In addition, a search of genome sequences of *B. cereus* strains in the National Center for Biotechnology Information (https://www.ncbi.nlm.nih.gov/) database showed that ST1420 had an allele profile closest to *B. cereus* strain 2M5. ST1420 and *B. cereus* strain 2M5 had the same allele numbers at 6 loci (*glpF, gmk, ilvD, pta, pur*, and *tpi*), including the *tpi* allele 210 detected in this study.

**Table 5:** Allele profiles of ST1420 and similar sequence types of *Bacillus cereus*, Japan*

Strain	ST	Gene allele							Country of origin
		*glpF*	*gmk*	*ilvD*	*pta*	*pur*	*pycA*	*tpi*	
ST1420 strains found in this study	1420†	62	1	93	109	55	102	210‡	Japan
BC1	366	62	1	113	109	55	102	83	Japan
2M5	NA	62	1	93	109	55	37	210‡	Burkina Faso

*NA, not available; ST, sequence type. †ST found in this study. ‡New alleles in the Multilocus Sequencing Typing Database (https://pubmlst.org).

**Figure 2:**
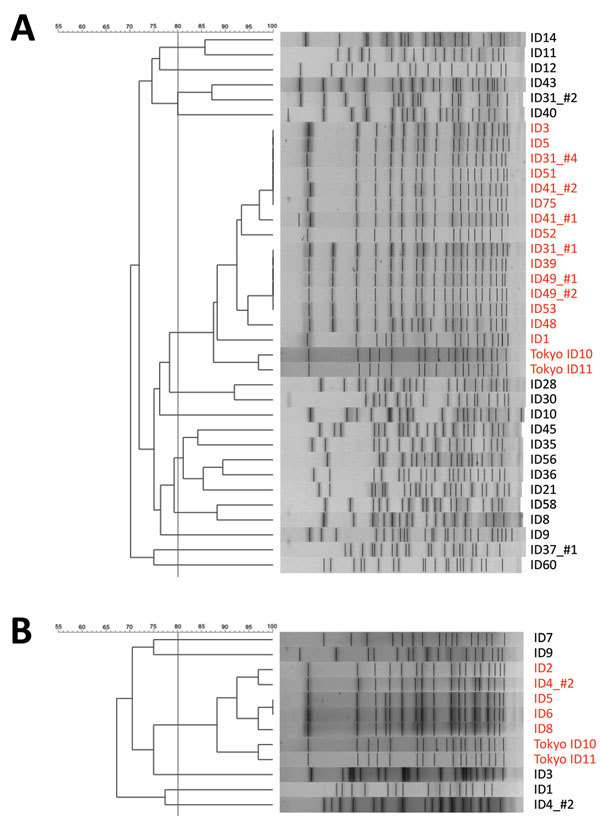
Pulsed-field gel electrophoresis (PFGE) results of the ST1420 strains of A) Tochigi strains and B) Tottori strains of *Bacillus cereus* isolates, Japan. The 80% similarity cutoff for PFGE cluster typing is shown as a vertical line in the phylogenetic tree. Names of strains of sequence type 1420 are indicated in red. The Tochigi_ID31_#3 was not analyzed. ID, identification. Scale bars indicate percent similarity.

To determine if ST1420 strains isolated from the different hospitals were derived from a single clone, we performed PFGE analysis for ST1420 strains from the Tochigi and Tottori strains and compared the results with those for Tokyo strains (Figure 2). All ST1420 strains formed a single cluster but some differences were observed in the band pattern. Therefore, although the ST is the same, these strains are not derived from a recently emerged single clone.

### Phylogenetic Analysis

**Figure 3:**
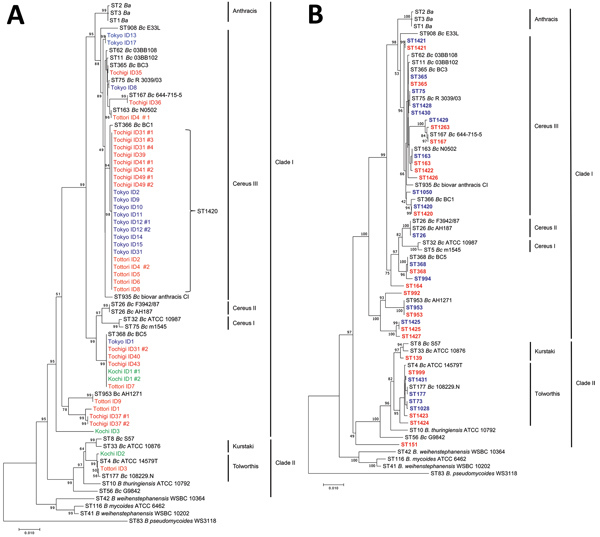
Multilocus sequence typing (MLST)–based phylogenetic trees of strains and STs of *Bacillus cereus* isolates, Japan. Reference sequences were obtained from the MLST database (https://pubmlst.org). Definitions of clades and lineage names followed those of Priest et al. (*9*). A) Phylogenetic tree of isolates from patients with bacteremia. Blue indicates Tokyo strains, red indicates Tochigi stains, orange indicates Tottori strains, and green indicates Kochi strains. B) Phylogenetic tree of STs detected in Tokyo and Tochigi strains. Blue indicates STs detected in Tokyo strains, and red indicates STs detected in Tochigi strains. Scale bars indicates nucleotide substitutions per site. Ba, *B. anthracis*; Bc, *B. cereus*; ID, identification; ST, sequence type.

To investigate the relationship between *B. cereus* isolates in the present study and other strains of the *B. cereus* group, we constructed phylogenetic trees by using concatenated sequences from 7 housekeeping genes used in MLST. ST1420 was classified into the Cereus III lineage, which is more closely related to the Anthracis lineage than to Cereus I and II lineages (Figure 3, panel A). Most strains isolated from patients with bacteremia were grouped into the Cereus III lineage, suggesting closer relationships with *B. anthracis*. Among Tokyo and Tochigi strains, isolates from patients with bacteremia were classified only into clade I, which includes Cereus I, Cereus II, Cereus III, and Anthracis lineages, whereas isolates from the environment were distributed not only in clade I but also in clade II (Figure 3, panel B).

## Discussion

We performed MLST analysis to identify genotypic characteristics of *B. cereus* clinical isolates from recent nosocomial infections in Japan. We established that ST1420, which has a novel combination of allele numbers of the 7 loci used in MLST, was the major ST in strains isolated from patients with bacteremia. The strains were isolated from hospitals in different prefectures of Japan in which nosocomial infections by *B. cereus* occurred during 2006, 2012, and 2016. Our analyses suggested that ST1420 is a high-risk clone that has a major association with recent nosocomial infections and bacteremia cases caused by *B. cereus* in Japan.

Tokyo and Tochigi are in the greater Tokyo area. However, the distance between the 2 hospitals in these cities is so large that nosocomial case-patients with the same *B. cereus* ST are rarely found. Nosocomial infections in Tochigi were associated with contaminated hospital linens (*14*). However, the linen suppliers are often quite localized, and it is unlikely that hospitals in Tochigi and Tokyo had the same suppliers. Although ST1420 was not found in Kochi strains, further studies are required to investigate the distribution of ST1420 clones in Japan by testing more strains of nosocomial infections in various places and years.

Phylogenetic relationships among reference strains of the *B. cereus* group showed that ST1420 strains were found in the Cereus III lineage. The Cereus III lineage is closely related to the Anthracis lineage, suggesting a close genetic background with *B. anthracis*. In a previous report, clinical isolates classified into the Cereus III lineage were also associated with systemic diseases (*11*); *B. cereus* 03BB102 (ST11), which was isolated from a patient who died from pneumonia, and *B. cereus* D4214 (ST62), which was isolated from a patient with septicemia, belonged to the Cereus III lineage. *B. cereus* strains isolated from other severe infection outbreaks in hospitals have been found to belong to the Cereus III lineage (*12*,*21*). The fact that ST1420, which is believed to be associated with bacteremia, belongs to the Cereus III lineage is consistent with the previously reported relationships between *B. cereus* strains causing severe symptoms.

To determine relationships between *B. cereus* strains belonging to the Cereus III lineage and *B. anthracis*, we analyzed isolates from our study for a genetic marker for *B. anthracis*. Ba813 is a 277-bp chromosomal DNA sequence present in *B. anthracis* and has been used for differentiation of *B. anthracis* from *B. cereus* (*22*). However, it has been reported that some *B. cereus* strains contain Ba813 (*12*,*23*). We found that some of the *B. cereus* isolates in our study were in the Cereus III lineage, including ST1420, contained Ba813. These results indicate close relationships among some *B. cereus* strains belonging to the Cereus III lineage with *B. anthracis* and suggest that Ba813 is not a suitable chromosomal genetic marker for identification of *B. anthracis*.

For other STs, ST167, ST365, and ST368 were detected in bacteremia cases in Tochigi. ST368 was also detected in strains isolated from patients with bacteremia in Tokyo, Tottori, and Kochi. ST167 has been reported in patients with bacteremia, ST365 has been reported in patients with sepsis, and ST368 has been reported in patients with pyrexia in Japan (*13*,*24*). ST365 and ST368 have been reported in a person with a nosocomial infection (*13*). In previous studies, some pathogenic *B. cereus* isolates associated with emetic illness were classified as ST26, and some isolates associated with pneumonia were classified as ST78 (*11*). Our findings suggest that specific STs are associated with nosocomial infections or severe infections.

In conclusion, we have shown that *B. cereus* ST1420, a novel ST, was a major ST among nosocomial infections and bacteremia cases in Japan that occurred in different hospitals and different years. ST1420 could be a prevalent ST in recent *B. cereus* nosocomial infections in Japan. Further investigations are required to elucidate its distribution.
